# CIRBP Stabilizes *Slc7a11* mRNA to Sustain the SLC7A11/GPX4 Antioxidant Axis and Limit Ferroptosis in Doxorubicin-Induced Cardiotoxicity

**DOI:** 10.3390/antiox14080930

**Published:** 2025-07-29

**Authors:** Yixin Xie, Yongnan Li, Yafei Xie, Jianshu Chen, Hong Ding, Xiaowei Zhang

**Affiliations:** 1 Department of Cardiology, Lanzhou University Second Hospital, Lanzhou 730000, China; 220220905070@lzu.edu.cn (Y.X.); xieyf20@lzu.edu.cn (Y.X.); chenjsh19@lzu.edu.cn (J.C.); dingh0110@163.com (H.D.); 2 The Second Clinical Medical College, Lanzhou University, Lanzhou 730000, China; lyngyq2006@foxmail.com; 3 Department of Cardiac Surgery, Lanzhou University Second Hospital, Lanzhou 730000, China

**Keywords:** CIRBP, doxorubicin, oxidative stress, cardiotoxicity, ferroptosis, SLC7A11, GPX4, RNA-binding protein, cardioprotection

## Abstract

Doxorubicin-induced cardiotoxicity (DIC) significantly constrains the clinical efficacy of anthracycline chemotherapy, primarily through the induction of ferroptosis, an iron-dependent, regulated cell death driven by oxidative stress and lipid peroxidation. However, the upstream regulators of ferroptosis in DIC remain incompletely defined. Cold-inducible RNA-binding protein (CIRBP) exhibits cardioprotective effects in various pathological contexts, but its precise role in ferroptosis-related cardiotoxicity is unknown. This study investigated whether CIRBP mitigates DIC by modulating the ferroptosis pathway via the SLC7A11 (Solute carrier family 7 member 11)/GPX4 (Glutathione peroxidase 4) axis. We observed marked downregulation of CIRBP in cardiac tissues and cardiomyocytes following doxorubicin exposure. CIRBP knockout significantly exacerbated cardiac dysfunction, mitochondrial damage, oxidative stress, and lipid peroxidation, accompanied by increased mortality rates. Conversely, CIRBP overexpression alleviated these pathological changes. Molecular docking and dynamics simulations, supported by transcriptomic analyses, revealed direct binding of CIRBP to the 3′-UTR of *Slc7a11* mRNA, enhancing its stability and promoting translation. Correspondingly, CIRBP deficiency markedly suppressed SLC7A11 and GPX4 expression, impairing cystine uptake, glutathione synthesis, and antioxidant defenses, thus amplifying ferroptosis. These ferroptotic alterations were partially reversed by ferroptosis inhibitor ferrostatin-1 (Fer-1). Collectively, this study identifies CIRBP as a critical regulator of ferroptosis in DIC, elucidating a novel post-transcriptional mechanism involving *Slc7a11* mRNA stabilization. These findings offer new insights into ferroptosis regulation and highlight CIRBP as a potential therapeutic target for preventing anthracycline-associated cardiac injury.

## 1. Introduction

Doxorubicin (DOX) is an extensively utilized anthracycline chemotherapeutic agent, effectively treating various cancers, including solid tumors, acute leukemia, lymphoma, and breast cancer [1]. Despite its widespread clinical use, DOX-induced cardiotoxicity (DIC) significantly constrains its therapeutic applications [1,2]. This cardiotoxicity, characterized by progressive and irreversible myocardial injury, can lead to severe clinical outcomes such as arrhythmias, pathological hypertrophy, ventricular dysfunction, and congestive heart failure, even years after treatment cessation [3,4]. Therefore, addressing DIC while preserving DOX’s anticancer efficacy remains a critical and unresolved clinical challenge.

Among the diverse mechanisms proposed for DIC, ferroptosis—a regulated, iron-dependent form of cell death driven by lipid peroxidation—has recently emerged as a pivotal factor [5,6,7]. Ferroptosis disrupts iron homeostasis and enhances lipid peroxidation, contributing significantly to myocardial damage and dysfunction [8,9]. Central to the regulation of ferroptosis is the SLC7A11 (Solute carrier family 7 member 11)/GPX4 (Glutathione peroxidase 4) pathway. SLC7A11 imports cystine into cells to sustain glutathione (GSH) synthesis, while GPX4 utilizes GSH to detoxify lipid peroxides, thereby protecting cardiomyocytes from oxidative injury and ferroptotic death [10,11,12].

Cold-inducible RNA-binding protein (CIRBP) is a stress-responsive RNA-binding protein induced by various cellular stressors, including hypoxia, ultraviolet radiation, reactive oxygen species, and cold shock [13,14]. Previous studies have demonstrated the protective effects of CIRBP in cardiomyocytes under multiple pathological conditions, primarily attributed to its role in regulating antioxidant capacity and apoptosis pathways [15]. Recent findings also suggest that CIRBP could influence cellular redox balance and ferroptosis through modulation of antioxidant genes like SLC7A11 and GPX4. For example, Shimizu et al. recently demonstrated extracellular CIRBP’s role in promoting GPX4-mediated ferroptosis [16,17].

Despite the growing evidence linking CIRBP to cellular stress responses, its specific regulatory role in DIC-related ferroptosis remains largely unknown. Current research into ferroptosis-associated cardiotoxicity primarily utilizes in vitro models and lacks comprehensive validation through in vivo and detailed molecular mechanisms [18]. Furthermore, direct interactions between CIRBP and ferroptosis pathways, specifically involving SLC7A11/GPX4, have not been explored comprehensively.

**Figure 1:**
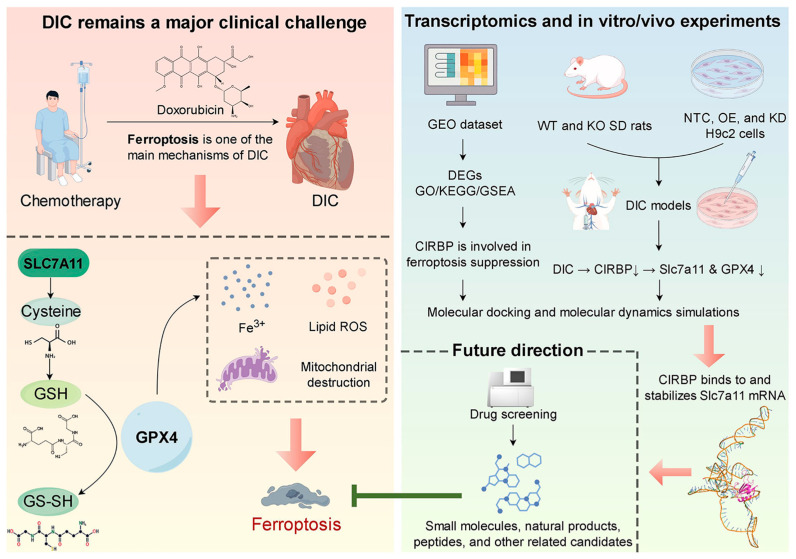
Prospect overview. Doxorubicin chemotherapy precipitates DIC by activating ferroptosis through suppression of the SLC7A11/GPX4 antioxidant axis. Transcriptomic profiling, together with complementary in vitro and in vivo experiments, demonstrates that CIRBP directly binds to and stabilizes *Slc7a11* mRNA, thereby sustaining SLC7A11/GPX4 expression and limiting ferroptosis. Future high-throughput screens will seek small-molecule, natural-product, and peptide compounds that up-regulate CIRBP activity to achieve cardioprotection. Black arrows indicate activation/positive regulation; green T-ended lines indicate inhibition; red arrows denote next steps/future directions. By Figdraw.

Based on these critical gaps, we hypothesize that CIRBP mitigates DIC by attenuating ferroptosis through direct interaction and regulation of the SLC7A11/GPX4 pathway. To address this hypothesis, our study systematically employs combined in vivo and in vitro models, complemented by advanced molecular docking and dynamic simulation techniques. By clearly delineating CIRBP’s regulatory mechanism in ferroptosis, our study presents a novel mechanistic insight into chemotherapy-associated cardiotoxicity and establishes CIRBP as a potential therapeutic target. Ultimately, our findings contribute significantly to understanding ferroptosis in cardiovascular diseases, potentially paving the way for innovative therapeutic strategies that minimize adverse cardiac effects without compromising anticancer efficacy (Figure 1).

## 2. Materials and Methods

Detailed methods are available in the Supplementary Methods.

### 2.1. Ethical Statement

All animal protocols complied with the Lanzhou University Second Hospital (LUSH) Guidelines for the Care and Use of Laboratory Animals and received formal clearance from the LUSH Institutional Animal Ethics Committee (approval No. D2024-191, 6 March 2024).

### 2.2. Animals

Male Sprague–Dawley rats and CIRBP-null littermates were randomly separated into four groups: (i) control, intraperitoneal (i.p.) saline; (ii) DOX, single i.p. dose of doxorubicin (15 mg kg^−1^; HY-15142, MedChemExpress, Monmouth Junction, NJ, USA); (iii) DOX + Fer-1, Ferrostatin-1 (HY-100579, MedChemExpress, Monmouth Junction, NJ, USA) administered i.p. 24 h before DOX followed by DOX, as above; (iv) Fer-1 alone. Baseline and post-treatment cardiac performance were quantified by echocardiography, after which animals were euthanized and hearts plus sera were harvested for histology, ultrastructure, and molecular assays.

### 2.3. Echocardiography

Under isoflurane anesthesia, two-dimensional and M-mode images at papillary–muscle level were captured with a Vevo 2100 (FUJIFILM VisualSonics, Toronto, ON, Canada); heart rate and left-ventricular dimensions were analyzed with integrated software.

### 2.4. Histological Analysis

Fixed hearts (4% paraformaldehyde) were paraffin-embedded, sectioned, and stained with H&E, WGA, and Masson’s trichrome; TUNEL identified apoptotic nuclei. For fluorescence, cells fixed in 4% paraformaldehyde were permeabilized (0.5% Triton X-100), blocked, incubated with primary antibodies, and visualized on an Olympus fluorescence microscope; Image-Pro Plus 6.0 quantified signal intensity.

### 2.5. Transmission Electron Microscopy

Myocardial tissue samples (≈1 mm^3^) was fixed in 2.5% glutaraldehyde, embedded, sectioned, and examined with a Hitachi TEM at 80 kV; mitochondrial injury was graded via the Flameng scale across ten mitochondria in five random fields per sample.

### 2.6. Cell Cultures and In Vitro Treatment

H9c2 cardiomyoblasts (FH-1004, FuHeng Biotechnology, Shanghai, China) were cultured at 37 °C with 5% CO_2_. After 48 h, plasmids, siRNAs, or control vectors were transfected; cells were then exposed to DOX, erastin (HY-15763, MedChemExpress, Monmouth Junction, NJ, USA), zVAD-fmk (M3143, AbMole BioScience, Houston, TX, USA), necrostatin-1 (SC4359, Beyotime, Shanghai, China), or 3-methyladenine (M2296, AbMole BioScience, Houston, TX, USA) at indicated concentrations.

### 2.7. Cell Counting and Viability Assay

Cells seeded at 5 × 10^3^ well^−1^ in 96-well plates were treated for 24 h, then incubated with CCK-8 reagent (C0038, Beyotime, Shanghai, China) for 2–3 h at 37 °C; absorbance at 450 nm was recorded using an EnSpire reader (PerkinElmer, Waltham, MA, USA).

### 2.8. Laser Confocal Detection of Mitochondrial Membrane Potential

JC-1 staining (E-CK-A301, Elabscience, Wuhan, China) in confocal dishes was performed following the manufacturer’s protocol; fluorescence was imaged with excitation at 488 nm and emissions at 525 nm (monomer) and 585 nm (aggregate).

### 2.9. Measurement of CIRBP, Markers of Myocardial Injury, and Ferroptosis

Rat serum CIRBP was measured using an ELISA kit (ml025286, mlBio, Shanghai, China). Myocardial injury markers, including BNP (CSB-E07972r, Cusabio, Wuhan, China), CK (YS01S62, Y-S Biotechnology, Beijing, China), cTnT (ml003374, mlBio, Shanghai, China), and LDH (ml092995, mlBio, Shanghai, China), were assessed using both ELISA and biochemical kits. Ferroptosis markers, including SOD (ml077379, mlBio, Shanghai, China), MDA (ml077384, mlBio, Shanghai, China), GSH (ml095262, mlBio, Shanghai, China), and Fe^2+^ (E-BC-F101, Elabscience, Wuhan, China), were also measured using a combination of ELISA and biochemical kits, depending on the marker.

### 2.10. Flow Cytometry and Imaging Flow Cytometry

Reactive oxygen species were detected with DCFH-DA (E-BC-K138-F, Elabscience, Wuhan, China) on a BD FACSCanto^TM^ II flow cytometer (BD Biosciences, San Jose, CA, USA), and data were processed in FlowJo (FlowJo LLC, Ashland, OR, USA).

### 2.11. Western Blot Analyses

Protein extracts were resolved by SDS-PAGE, transferred to nitrocellulose, and probed with target-specific antibodies; GAPDH served as loading control.

### 2.12. Quantitative Real-Time PCR

Total RNA was isolated from rat tissues or cultured cells. Complementary DNA (cDNA) was synthesized for reverse transcription-quantitative polymerase chain reaction (RT-qPCR).

### 2.13. Datasets and Bioinformatic Analysis

GEO2R analyzed differential CIRBP and SLC7A11 expression in GEO datasets; RNA-binding predictions employed starBase and RBPDB; single-cell and proteomic data were retrieved from the Human Protein Atlas; functional enrichment (GO, KEGG) used DAVID.

### 2.14. Molecular Docking and Molecular Dynamics Simulations

The interaction between CIRBP and the 3′-UTR region of *Slc7a11* mRNA was analyzed using molecular docking and molecular dynamics (MD) simulations. CIRBP was modeled using AlphaFold Protein Structure Database (version 2.3.1), and RNAfold (ViennaRNA Package, version 2.5.1) predicted the *Slc7a11* mRNA structure. AutoDock Vina (version 1.2.3) was used for docking to identify binding sites, followed by 100 ns MD simulations using GROMACS (version 2021.4) to assess complex stability. Key interaction residues and stability metrics, including RMSD and RMSF, were analyzed to elucidate the molecular basis of CIRBP’s regulation of *Slc7a11* mRNA.

### 2.15. Statistical Analysis

GraphPad Prism 9.5.1 analyzed data expressed as mean ± SD. Two-group comparisons employed Student’s *t*-test or Mann–Whitney U test; multi-group differences were assessed with one- or two-way ANOVA followed by Dunnett’s or Šídák’s post hoc tests. Survival curves were compared by log-rank analysis, and repeated-measures ANOVA evaluated longitudinal data; significance was accepted at *p* < 0.05.

## 3. Results

### 3.1. CIRBP Expression Is Downregulated in DIC

**Figure 2:**
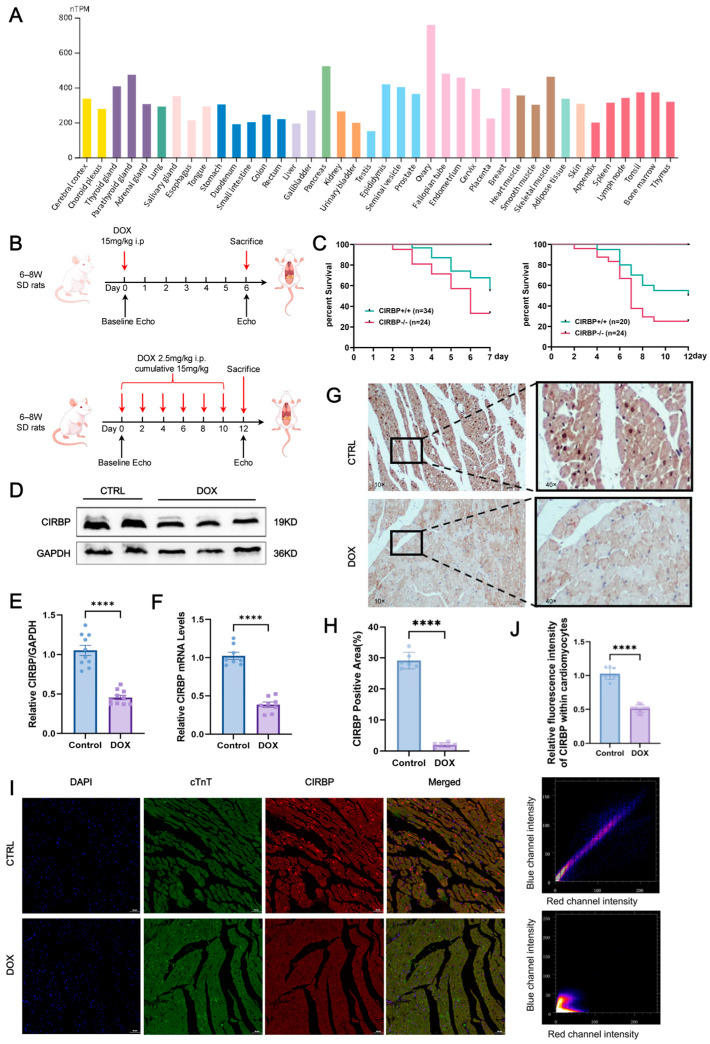
CIRBP is broadly expressed in tissues and is downregulated in DIC. (**A**) Expression levels of CIRBP across 37 rat tissues based on RNA-seq data retrieved from the Human Protein Atlas. The heart shows medium-to-high expression, visualized as normalized transcripts per million (nTPM). (**B**) Schematic illustration of experimental protocols for DIC in SD rats. Two regimens were used as follows: an acute model (single intraperitoneal injection of 15 mg/kg) and a chronic model (2.5 mg/kg × 6 doses, cumulative 15 mg/kg). The chronic model (highlighted in red) was used in subsequent experiments to better mimic clinical DIC. (**C**) Kaplan–Meier survival curves comparing wild-type (WT) and CIRBP^−/−^ rats under acute and chronic DOX treatment conditions. CIRBP deficiency was associated with significantly reduced survival (*n* = 20–34/group). (**D**–**F**) Representative Western blot images and quantitative analysis of CIRBP levels (**D**,**E**) and *Cirbp* mRNA levels (**F**) in cardiac tissues from control and DOX groups (*n* = 8). (**G**) Representative immunohistochemical (IHC) images and quantification showing a marked reduction in CIRBP-positive area in the DOX group (*n* = 6, scale bars = 50 µm). (**H**,**I**) Immunofluorescence (IF) staining for CIRBP (red), cardiac troponin T (cTnT, green), and nuclei (DAPI, blue) in control and DOX groups. In the control group, CIRBP was primarily localized in cardiomyocyte nuclei, while in the DOX group, CIRBP expression was significantly decreased and shifted toward the cytoplasm. Arrows indicate CIRBP-positive regions (*n* = 6, scale bars = 50 µm). (**J**) Quantification of CIRBP fluorescence intensity within cardiomyocytes, confirming significant reduction in the DOX group. Data are presented as mean ± SEM. Statistical significance was assessed using unpaired two-tailed Student’s *t*-test (**A**,**C**) or one-way ANOVA with Tukey’s multiple comparisons test (**D**–**J**). **** *p* < 0.0001.

Mining of the Human Protein Atlas RNA-seq database revealed that CIRBP transcripts are constitutively expressed at medium-to-high levels in rat myocardium (Figure 2A). This suggests that CIRBP may play an essential role in maintaining cardiac homeostasis. To investigate the association between CIRBP and DIC, we generated two Sprague–Dawley rat models: an acute injury model (single intraperitoneal dose, 15 mg kg^−1^, evaluated on day 6) and a chronic injury model (cumulative 15 mg kg^−1^, delivered as 2.5 mg kg^−1^ × 6 over 12 days) (Figure 2B). Functional and survival read-outs demonstrated that the chronic regimen most faithfully reproduced the progressive myocardial injury seen clinically and was thus adopted for subsequent mechanistic experiments (Figure 2C).

Both Western blotting and RT-qPCR confirmed a pronounced reduction in CIRBP and *Cirbp* mRNA in the DOX group compared with controls (Figure 2D–F). Immunohistochemistry showed a marked decrease in CIRBP-positive area across the ventricular wall (Figure 2G,H), and dual immunofluorescence staining demonstrated that CIRBP was predominantly localized in the nuclei of cardiomyocytes under physiological conditions. In the DOX group, CIRBP expression was markedly reduced, and its subcellular distribution shifted toward the cytoplasm (Figure 2I,J).

Collectively, these results demonstrate that CIRBP expression is significantly downregulated in the hearts of rats from the DOX group, suggesting a potential involvement of CIRBP in the pathological process of DIC.

### 3.2. CIRBP Deletion Aggravates DIC In Vivo

**Figure 3:**
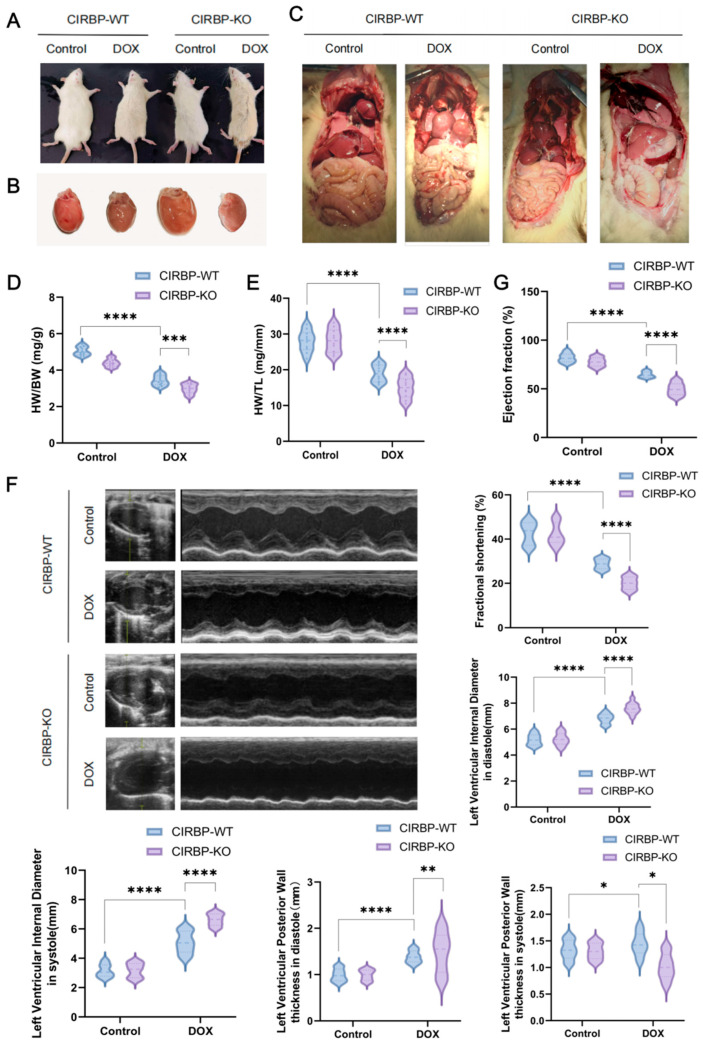
CIRBP deficiency exacerbates doxorubicin-induced cardiac dysfunction. (**A**) Representative images showing general appearance of CIRBP-WT and CIRBP-KO groups following doxorubicin (DOX) treatment. (**B**) Gross morphology of hearts from CIRBP-WT and CIRBP-KO groups, with visible cardiac atrophy in the CIRBP-KO DOX group. (**C**) Representative images of internal organs demonstrating systemic changes associated with DOX-induced cardiotoxicity. (**D**,**E**) Quantification of heart-weight-to-body-weight (HW/BW) and heart-weight-to-tibia-length (HW/TL) ratios. (**F**) Representative M-mode echocardiographic images and quantitative analysis of left ventricular (LV) internal diameters during systole and diastole, and posterior wall thickness in both phases. (**G**) Indicators such as left ventricular ejection fraction (LVEF) and fractional shortening (FS) were measured to assess systolic function. All data are presented as mean ± SEM (*n* = 6–8/group). Statistical comparisons were performed using one-way ANOVA followed by Tukey’s post hoc test. * *p* < 0.05, ** *p* < 0.01, *** *p* < 0.001, **** *p* < 0.0001.

To investigate the functional role of CIRBP in DIC, we compared the phenotypes of CIRBP-WT and CIRBP-KO group subjected to DOX treatment. Gross morphological inspection revealed more severe systemic and cardiac changes in the CIRBP-KO DOX group, including pericardial effusion, pleural edema, and hepatic congestion (Figure 3A–C). Hearts from CIRBP-KO group in the DOX group exhibited pronounced atrophy and pallor, indicative of aggravated myocardial damage. Cardiac morphometric indices showed significant worsening in the CIRBP-KO group. Both heart-weight-to-body-weight (HW/BW) and heart-weight-to-tibia-length (HW/TL) ratios were significantly increased in the DOX-treated CIRBP-KO group compared to DOX-treated CIRBP-WT group (Figure 3D,E).

Echocardiographic analysis confirmed that CIRBP deletion significantly exacerbated DOX-induced cardiac dysfunction (Figure 3F). Left ventricular ejection fraction (EF) and fractional shortening (FS) were markedly lower in CIRBP-KO DOX rats compared to CIRBP-WT DOX rats (Figure 3G). Furthermore, left ventricular internal diameters during diastole and systole were significantly increased in CIRBP-KO DOX rats, indicating chamber dilation. Posterior wall thickness in diastole and systole was also significantly reduced in the absence of CIRBP, consistent with myocardial thinning and compromised structural integrity.

Together, these findings demonstrate that CIRBP deficiency markedly aggravates structural and functional deterioration in the heart under DIC conditions, highlighting a protective role for CIRBP in maintaining myocardial integrity during chemotherapeutic stress.

### 3.3. CIRBP Provides Cardioprotection Against DIC In Vivo

**Figure 4:**
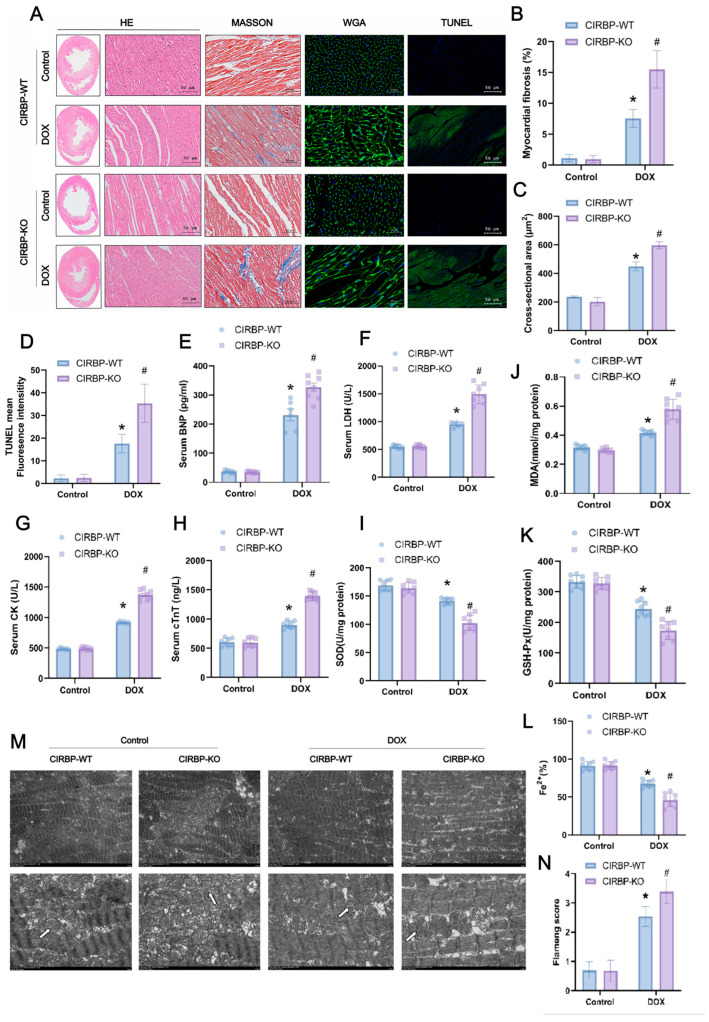
CIRBP knockout exacerbates DOX-induced cardiotoxicity. (**A**) Histological analysis of cardiac tissues from CIRBP-WT and CIRBP-KO groups in control and DOX-treated groups using hematoxylin and eosin (H&E), Masson’s trichrome, wheat germ agglutinin (WGA), and TUNEL staining. Masson’s trichrome staining highlights increased myocardial fibrosis in the CIRBP-KO group treated with DOX. (**B**) Quantification of myocardial fibrosis percentage from Masson’s trichrome staining. (**C**) Cross-sectional area of cardiomyocytes measured from WGA-stained sections, indicating increased hypertrophy in the CIRBP-KO group. (**D**) Quantification of TUNEL-positive fluorescence intensity, showing increased apoptosis in the CIRBP-KO group. (**E**–**H**) Serum levels of BNP, LDH, CK, and cTnT in CIRBP-WT and CIRBP-KO groups, indicating increased myocardial injury in the CIRBP-KO group treated with DOX. (**I**–**L**) Assessment of oxidative stress markers, including MDA, SOD, GSH-Px, and Fe^2+^ content. (**M**,**N**) Transmission electron microscopy images showing mitochondrial morphology in each group (arrows indicate selected mitochondria used for morphological comparison between groups) Flameng scoring for mitochondrial ultrastructural damage. Data are presented as mean ± SD. Statistical analysis by unpaired Student’s *t*-test, * *p* < 0.05, # *p* < 0.01.

To further assess the role of CIRBP in DIC, we evaluated myocardial structural integrity, apoptosis, and oxidative stress markers in CIRBP-WT and CIRBP-KO rats. Histological analyses (Figure 4A) revealed that DOX-treated CIRBP-KO hearts exhibited aggravated pathological changes, including extensive myofiber disarray (H&E), increased interstitial fibrosis (Masson), enlarged cardiomyocyte cross-sectional area (WGA), and elevated apoptosis (TUNEL). Quantification confirmed significantly greater myocardial fibrosis (Figure 4B), cardiomyocyte hypertrophy (Figure 4C), and TUNEL-positive fluorescence intensity (Figure 3D) in the CIRBP-KO DOX group compared to CIRBP-WT. Serum markers of myocardial injury were markedly elevated in CIRBP-KO rats after DOX treatment. Circulating levels of B-type natriuretic peptide (BNP), lactate dehydrogenase (LDH), creatine kinase (CK), and cardiac troponin T (cTnT) were all significantly increased in the CIRBP-KO DOX group compared to CIRBP-WT controls (Figure 4E–H), indicating exacerbated myocardial stress and cell damage.

Consistent with these findings, oxidative stress was more pronounced in CIRBP-KO hearts under DOX challenge. Malondialdehyde (MDA) levels were significantly elevated, while antioxidant enzymes such as superoxide dismutase (SOD) and glutathione peroxidase (GSH-Px) were significantly decreased in CIRBP-KO rats (Figure 4I–K). Moreover, tissue Fe^2+^ levels were lower in CIRBP-KO hearts, suggesting disrupted iron homeostasis (Figure 4L). Transmission electron microscopy revealed substantial mitochondrial structural damage in the CIRBP-KO DOX group, including cristae fragmentation and vacuolar degeneration (Figure 4M). These pathological alterations were reflected in a significantly higher mitochondrial Flameng score (Figure 4N). Collectively, these data indicate that CIRBP deficiency aggravates DOX-induced myocardial oxidative stress, iron dysregulation, and ultrastructural damage, underscoring the antioxidant and cytoprotective role of CIRBP in the heart.

### 3.4. CIRBP Is Involved in Doxorubicin-Induced Ferroptosis

**Figure 5:**
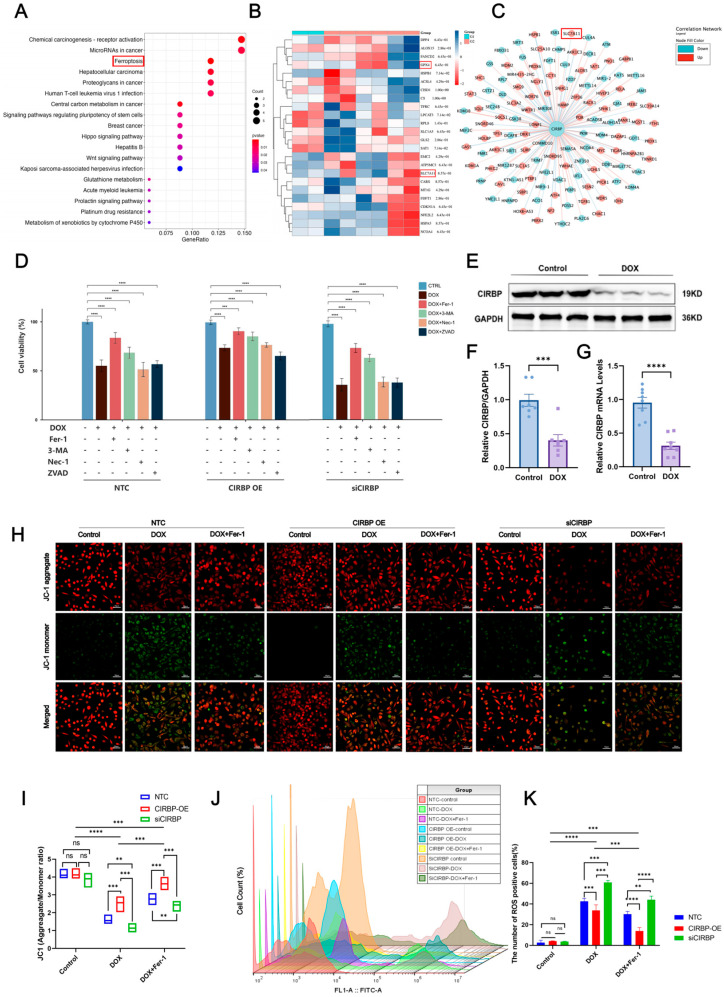
Bioinformatic and experimental analysis of CIRBP-regulated ferroptosis in DOX-induced cardiotoxicity. (**A**) GO and KEGG enrichment analyses of differentially expressed genes (DEGs) from the GSE218397 dataset reveal significant enrichment in ferroptosis-related pathways. (**B**) Heatmap showing reduced expression of canonical ferroptosis regulators SLC7A11 and GPX4 in DOX-treated cardiomyocytes. (**C**) Co-expression network analysis identifies CIRBP as a potential upstream regulator of ferroptosis-related genes including SLC7A11 and GPX4. (**D**) Cell viability assays indicate that among various cell death pathways, inhibition of ferroptosis (by Ferrostatin-1) most effectively restores viability in DOX-treated cells. CIRBP overexpression enhances, while CIRBP knockdown reduces, the protective effect of Fer-1 (*n* = 6). (**E**–**G**) Western blot and RT-qPCR analyses confirm significant downregulation of CIRBP and *Cirbp* mRNA expression in cardiomyocytes following DOX exposure (*n* = 6). (**H**,**I**) JC-1 staining reveals that CIRBP overexpression preserves mitochondrial membrane potential under DOX treatment, whereas CIRBP knockdown exacerbates mitochondrial depolarization. Quantification of the JC-1 aggregate/monomer ratio reflects these mitochondrial changes (*n* = 6). (**J**) Flow cytometry histogram overlays show intracellular ROS levels across treatment groups. (**K**) Quantification of ROS-positive cell percentages indicates that CIRBP knockdown significantly increases DOX-induced ROS production, while CIRBP overexpression reduces ROS accumulation. Ferrostatin-1 reverses ROS elevation (*n* = 6). Data are presented as mean ± SEM. *ns*, not significant; ** *p* < 0.01, *** *p* < 0.001, **** *p* < 0.0001 versus control; Scale bars: 50 μm.

To explore the regulatory role of CIRBP in ferroptosis, transcriptomic analysis and KEGG enrichment were performed. As shown in the enrichment plot, ferroptosis was one of the significantly enriched pathways in DOX-treated cardiomyocytes (Figure 5A). Heatmap and co-expression network analysis further identified key ferroptosis-related genes, including GPX4 and SLC7A11, to be downregulated upon DOX exposure (Figure 5B,C).

We next evaluated the effect of CIRBP on cell death under DOX treatment using multiple inhibitors targeting ferroptosis (Fer-1), autophagy (3-MA), necroptosis (Nec-1), and apoptosis (Z-VAD). The results revealed that Fer-1 partially rescued DOX-induced cell death in all groups, with CIRBP overexpression improving cell viability, while CIRBP knockdown exacerbated cell injury (Figure 5D). Western blot and RT-qPCR validated that CIRBP expression was significantly reduced after DOX treatment (Figure 5E–G), consistent with prior in vivo observations. JC-1 staining indicated that DOX disrupted mitochondrial membrane potential (MMP). CIRBP overexpression preserved MMP, while siCIRBP aggravated mitochondrial depolarization, which was partially reversed by Fer-1 (Figure 5H,I).

Flow cytometry further quantified ROS levels, showing that CIRBP knockdown significantly increased ROS-positive cardiomyocytes following DOX treatment. In contrast, CIRBP overexpression reduced ROS accumulation (Figure 5J,K). Collectively, these findings suggest that CIRBP mitigates DOX-induced ferroptosis by maintaining mitochondrial integrity and attenuating oxidative stress.

### 3.5. CIRBP Modulates Ferroptosis by Regulating the SLC7A11/GPX4 Axis In Vivo

**Figure 6:**
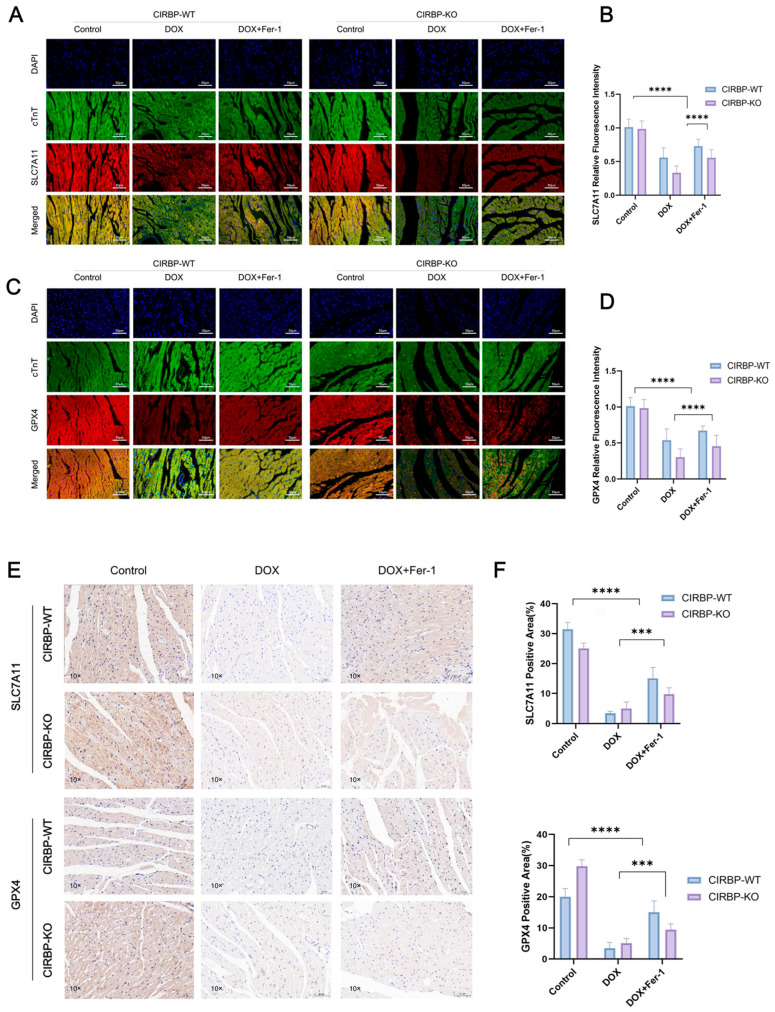
CIRBP inhibits ferroptosis in vivo by regulating the SLC7A11/GPX4 axis. (**A**,**B**) Representative immunofluorescence images of SLC7A11 (red), cTnT (green), and DAPI (blue) in myocardial tissues from CIRBP-WT and CIRBP-KO rats across Control, DOX, and DOX+Fer-1 groups, and corresponding quantification of relative fluorescence intensity (*n* = 6). (**C**,**D**) Representative immunofluorescence images of GPX4 (red), cTnT (green), and DAPI (blue), and corresponding quantification of GPX4 relative fluorescence intensity in the same experimental groups (*n* = 6). (**E**,**F**) Representative immunohistochemical staining of SLC7A11 and GPX4 in myocardial sections and quantification of positive area percentage (% of total tissue area) (*n* = 6). Data are shown as mean ± SEM. Statistical analysis was performed using two-way ANOVA followed by Tukey’s multiple comparisons test. *** *p* < 0.001, **** *p* < 0.0001.

To validate the regulatory role of CIRBP in ferroptosis-related pathways in vivo, we assessed the expression of key ferroptosis suppressors SLC7A11 and GPX4 in myocardial tissues from CIRBP-WT and CIRBP-KO rats subjected to DOX and/or ferrostatin-1 (Fer-1) treatment. Immunofluorescence staining revealed significant downregulation of both SLC7A11 and GPX4 in the DOX-treated CIRBP-WT group, while CIRBP deficiency further reduced their expression under the same conditions (Figure 6A–D). Quantification of fluorescence intensity confirmed that CIRBP-KO rats exhibited markedly lower SLC7A11 and GPX4 levels following DOX exposure compared to WT controls (*p* < 0.0001), and this repression was reversed by Fer-1 treatment. Consistently, immunohistochemical analysis of ventricular tissue sections showed a significant reduction in SLC7A11- and GPX4-positive areas in DOX-treated CIRBP-WT hearts, which was further diminished in CIRBP-KO animals (Figure 6E). Quantitative analysis demonstrated a CIRBP-dependent modulation of ferroptosis-related protein levels in vivo (Figure 6F). These results strongly suggest that CIRBP protects against DOX-induced cardiotoxicity by maintaining the expression of SLC7A11 and GPX4, thereby suppressing ferroptotic cell death in myocardial tissue.

### 3.6. CIRBP Upregulates the SLC7A11/GPX4 Axis to Inhibit Ferroptosis In Vitro

**Figure 7:**
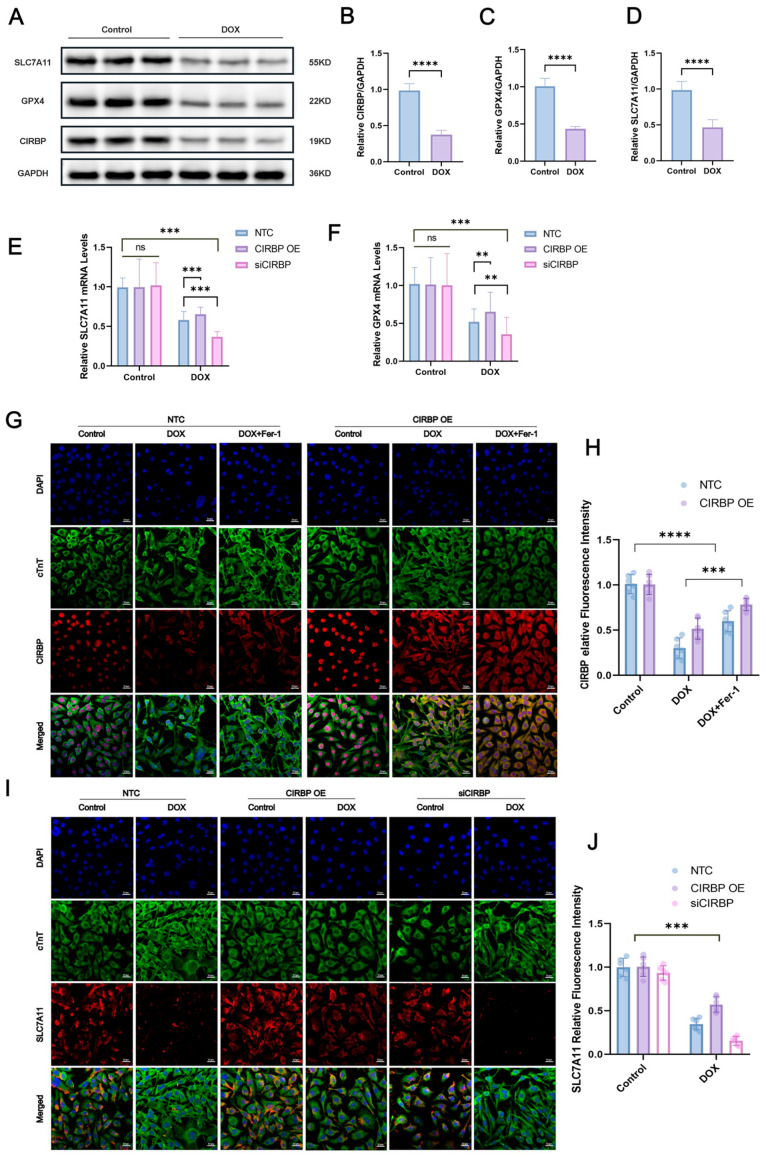
CIRBP attenuates DOX-induced ferroptosis in cardiomyocytes via the SLC7A11/GPX4 pathway. (**A**) Representative immunoblots of SLC7A11, GPX4, and CIRBP in control and DOX-treated H9c2 cells; GAPDH was the loading control. (**B**–**D**) Densitometric quantification of CIRBP, SLC7A11, and GPX4 protein abundance (*n* = 4 independent cultures). (**E**,**F**) RT–qPCR analysis of *Slc7a11* (**E**) and *Gpx4* (**F**) mRNA in non-target control (NTC), CIRBP-overexpression (CIRBP OE) and CIRBP-silenced (siCIRBP) cells under basal and DOX conditions (*n* = 6). (**G**) Immunofluorescence images showing nuclear DAPI (blue), cTnT (green) and CIRBP (red) in the indicated groups; scale bars = 20 μm. (**H**) Quantification of cytoplasmic CIRBP fluorescence intensity from panel G (*n* = 30 cells per group). (**I**) Immunofluorescence detection of SLC7A11 (red) together with cTnT (green) and DAPI (blue) in NTC, CIRBP OE, and siCIRBP cells ± DOX; scale bars = 20 μm. (**J**) Quantification of SLC7A11 fluorescence intensity from panel I (*n* = 30 cells per group). Data are expressed as mean ± SEM. Statistical significance was assessed by one-way ANOVA followed by Tukey’s post hoc test. *ns*, not significant, ** *p* < 0.01, *** *p* < 0.001, **** *p* < 0.0001; ns, not significant.

**Figure 8:**
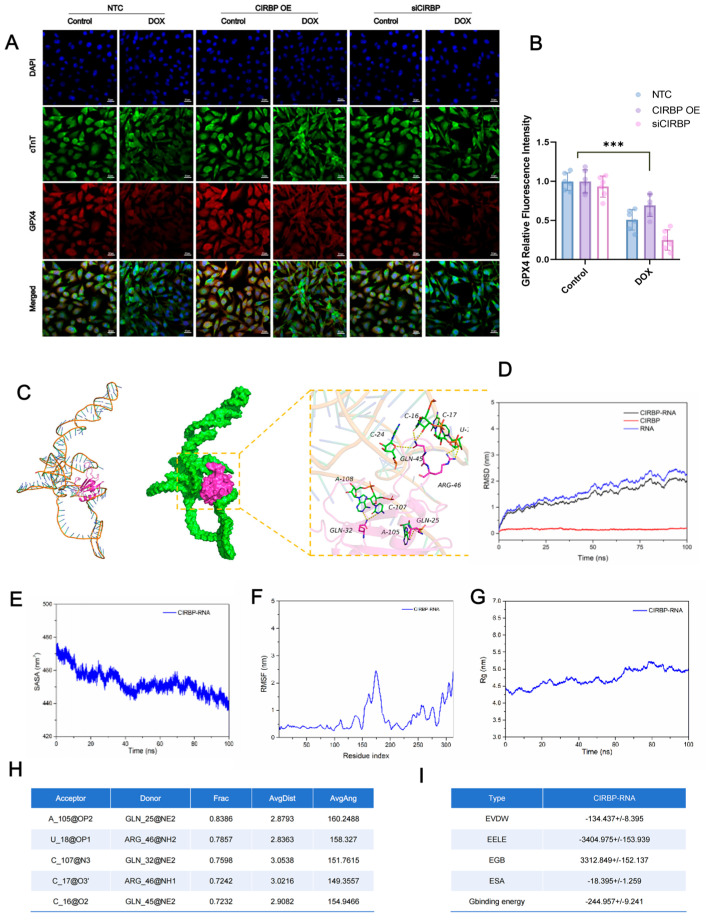
Molecular docking and dynamics of the CIRBP and *Slc7a11* mRNA interaction. (**A**) Immunofluorescence images of GPX4 (red), cTnT (green) and DAPI (blue) in NTC, CIRBP-overexpression (CIRBP OE), and CIRBP-silenced (siCIRBP) H9c2 cells with or without DOX; scale bars = 20 μm. (**B**) Quantification of GPX4 fluorescence intensity in panel A (*n* = 30 cells per group). (**C**) Molecular-docking model (left) and 100 ns molecular-dynamics (MD)–refined complex (middle) showing CIRBP (magenta) nested in the stem-loop of the *Slc7a11* 3′-UTR (green); enlarged view (right) depicts key hydrogen-bond contacts (black dashed lines) involving GLN-25, GLN-32, GLN-45, and ARG-46. (**D**) Root-mean-square deviation (RMSD) trajectories for CIRBP (red), RNA (blue) and the CIRBP—RNA complex (black) during MD simulation. (**E**) Solvent-accessible surface area (SASA) and (**F**) root-mean-square fluctuation (RMSF) plots reveal progressive complex compaction and limited backbone flexibility. (**G**) Radius of gyration (Rg) profile indicating gradual stabilization of the complex. (**H**) Summary of the five most persistent hydrogen bonds (occupancy > 70%; Frac) between CIRBP residues and *Slc7a11* RNA bases throughout the 100 ns trajectory. (**I**) MM/GBSA-binding free-energy components for the CIRBP–RNA complex (mean ± SD, kcal/mol); dominant contributions arise from electrostatic (EELE) and van der Waals (EVDW) interactions, yielding a favorable ΔG_binding of −244.96 ± 9.24 kcal/mol. Data are presented as mean ± SEM. Statistical analysis was performed by one-way ANOVA with Tukey’s post hoc test. *** *p* < 0.001; ns, not significant.

To investigate the regulatory role of CIRBP in ferroptosis, Western blot analysis (Figure 7A–D) demonstrated that doxorubicin (DOX) significantly downregulated CIRBP, SLC7A11, and GPX4 expression in cardiomyocytes. Immunofluorescence staining further confirmed that CIRBP overexpression restored CIRBP levels under DOX treatment, while siRNA-mediated knockdown markedly suppressed its expression (Figure 7E,F). Notably, CIRBP overexpression prevented the DOX-induced downregulation of SLC7A11 (Figure 7G–J) and GPX4 (Figure 8A,B), suggesting that CIRBP maintains redox homeostasis via the SLC7A11/GPX4 axis.

### 3.7. CIRBP Directly Interacts with Slc7a11 mRNA to Stabilize Its Expression

To explore the underlying mechanism of CIRBP-mediated regulation of SLC7A11, molecular docking and dynamics simulations were performed. Structural modeling showed stable binding between CIRBP and *Slc7a11* mRNA, with multiple hydrogen bonds involving residues such as GLN-25, ARG-46, and GLN-32 (Figure 8C). RMSD and Rg plots confirmed that the CIRBP–RNA complex maintained structural stability during the 100 ns simulation (Figure 8D–G). Furthermore, key hydrogen bonds persisted throughout the trajectory (Figure 8H), and binding free energy analysis revealed a favorable ΔG_binding of −244.96 kcal/mol, dominated by electrostatic and van der Waals interactions (Figure 8I). These findings suggest that CIRBP enhances *Slc7a11* mRNA stability via direct binding, thereby promoting its expression and ferroptosis resistance.

## 4. Discussion

DOX is widely used as a potent chemotherapeutic agent against various cancers, but its clinical utility is severely constrained by significant cardiotoxic side effects [1,2]. These adverse effects primarily arise from oxidative stress, mitochondrial dysfunction, and various regulated cell death pathways, including ferroptosis [5,6,7]. Ferroptosis, characterized by iron-dependent lipid peroxidation and compromised antioxidant defenses, has emerged as a key mechanism underlying DOX-induced cardiac injury [6,7,19,20]. In recent years, ferroptosis has been redefined as an integrated process involving multiple tissues and metabolic pathways. For example, gut microbiota metabolism can remotely regulate ferroptosis susceptibility in target organs by influencing host iron homeostasis and the supply of polyunsaturated lipid substrates. In pathological conditions such as neurodegeneration, tumor immunity, and cardiovascular injury, ferroptosis acts as a “double-edged sword”, accelerating tissue injury while facilitating tumor clearance; thus, precise spatial and temporal control is crucial. Mitochondria play dual roles in ferroptosis, serving not only as sources of iron-sulfur clusters and reactive oxygen species (ROS) but also as amplifiers or suppressors of ferroptosis through lipid remodeling and changes in membrane potential. Accordingly, antioxidant therapeutic strategies are evolving from simple ROS scavenging toward systemic modulation of iron-sulfur-lipid networks, including small-molecule iron chelation, transactivation of GPX4, and lipid metabolism reprogramming [21,22,23,24]. Collectively, iron-dependent lipid peroxidation, excessive ROS, and dynamic mitochondrial function constitute the core ferroptotic pathways, providing a theoretical foundation for identifying novel regulatory nodes (such as the CIRBP–SLC7A11/GPX4 axis) and their targeted interventions.

This study systematically elucidates the protective mechanism by which CIRBP regulates ferroptosis via the SLC7A11/GPX4 axis, thus attenuating DIC. Our findings significantly broaden the theoretical framework of anthracycline-induced cardiotoxicity and underscore the critical roles of RNA-binding proteins in cardiac homeostasis and pathology.

The marked reduction in CIRBP levels following DOX treatment indicates its potential involvement in cardiotoxic stress responses [10], aligning with previous studies identifying RNA-binding proteins as key regulators of cellular stress adaptation [25,26,27]. Earlier studies indicated that RNA-binding proteins manage mRNA dynamics during oxidative or inflammatory stress. Our study further links CIRBP specifically to ferroptosis, a regulated form of cell death driven by lipid peroxidation and compromised antioxidant defenses [14,15], offering novel theoretical insights into RNA-protein interactions under chemotherapeutic stress [28,29]. Detailed analyses demonstrated that CIRBP deficiency exacerbates DOX-induced cardiac injury, oxidative stress, and cardiac remodeling, reaffirming its critical protective role in maintaining cardiac structure and function, consistent with previous findings regarding RNA-binding proteins’ roles in stress resilience [17,30].

Intervention with apoptosis inhibitors, pyroptosis inhibitors, necroptosis inhibitors, and the ferroptosis inhibitor Fer-1 in cardiomyocytes treated with doxorubicin confirmed ferroptosis as the predominant form of DOX-induced cardiomyocyte death [18], establishing the specificity of ferroptosis in mediating cardiotoxicity [31]. The clear link between CIRBP expression and ferroptosis regulation via the SLC7A11/GPX4 pathway expands our theoretical understanding of cell-death modalities in cardiac pathology and positions ferroptosis as a promising therapeutic target for cardioprotection.

**Figure 9:**
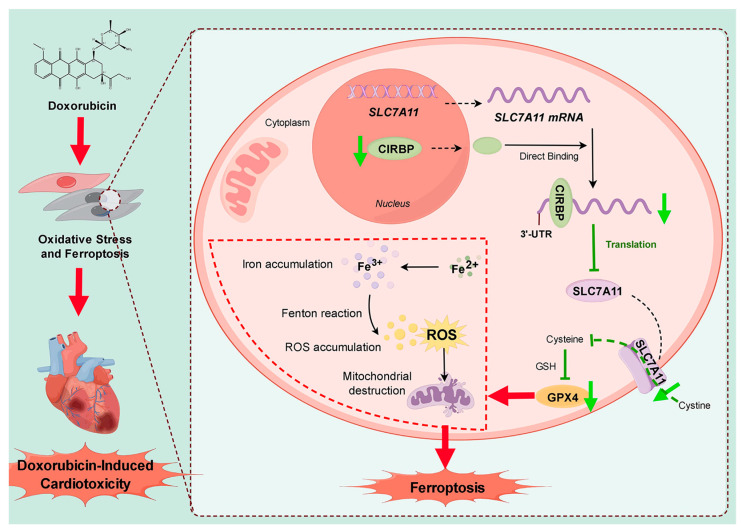
Schematic model summarizing the protective role of CIRBP against DIC. Doxorubicin (left) triggers oxidative stress and ferroptosis in cardiomyocytes, culminating in progressive myocardial injury. In the absence of stress (right panel), CIRBP binds directly to the 3′-UTR of *Slc7a11* mRNA, stabilizing the transcript and promoting its translation. The resulting SLC7A11 protein imports cystine, sustaining GSH synthesis and allowing GPX4 to detoxify lipid peroxides, thereby preventing iron accumulation, ROS overproduction, and mitochondrial damage. Doxorubicin downregulates CIRBP, reducing SLC7A11 and GPX4 expression, impairing cystine/GSH metabolism and unleashing ferroptosis, which drives DIC. Restoration of CIRBP would therefore re-establish the SLC7A11/GPX4 axis, limit ferroptosis, and preserve cardiac function. Black solid arrows indicate promotion; black dashed arrows indicate translocation; green arrows indicate downregulation; green T-shaped arrows indicate inhibition; red arrows indicate pathological processes. By Figdraw.

Mechanistically, doxorubicin treatment suppresses CIRBP expression, disrupting *Slc7a11* mRNA stability, thereby impairing the SLC7A11/GPX4 antioxidant axis. This impairment leads to intracellular iron (Fe^3+^) accumulation, excessive ROS generation, mitochondrial dysfunction, and ultimately ferroptosis-mediated cardiac damage. Conversely, restoration of CIRBP stabilizes *Slc7a11* mRNA and maintains antioxidant defense, highlighting the therapeutic potential of targeting the CIRBP–SLC7A11–GPX4 pathway to alleviate ferroptosis and preserve cardiac function. CIRBP protects mitochondrial function and redox balance by sustaining the expression of key ferroptosis suppressors. CIRBP overexpression restores mitochondrial membrane potential and reduces ROS accumulation, underscoring its critical role in mitochondrial homeostasis, a central node within ferroptosis pathways [32]. This mechanism aligns well with reports identifying mitochondrial dysfunction as a pivotal driver of ferroptosis and oxidative stress in cardiomyopathies [33,34,35]. Molecular docking analysis revealed direct interaction between CIRBP and *Slc7a11* mRNA, and molecular dynamics simulations identified the critical residues and structural motifs stabilizing the CIRBP–mRNA complex, clarifying how CIRBP promotes translation of antioxidant genes. Such RNA-binding activity presents novel regulatory mechanisms with therapeutic targeting potential (Figure 9).

Computational analyses revealed direct interactions between CIRBP and *Slc7a11* mRNA. Molecular docking and dynamics simulations identified critical residues and structural motifs responsible for stabilizing CIRBP–mRNA complexes, elucidating how CIRBP promotes the translation of antioxidant genes. Such RNA-binding activity provides novel regulatory mechanisms suitable for therapeutic targeting.

Compared with the existing literature, our study offers practical and theoretical innovations. Historically, research on anthracycline cardiotoxicity primarily emphasized apoptosis and general oxidative stress, with limited attention given to ferroptosis and RNA-binding proteins. This study explicitly identifies CIRBP as a central regulator of ferroptosis, introducing novel therapeutic targets. Future research should further validate CIRBP–RNA interactions through biochemical and genetic approaches to solidify the molecular mechanisms involved. Moreover, identifying additional RNA targets of CIRBP may expand our understanding of RNA-mediated regulation in cardiac pathology, potentially uncovering broader therapeutic applications. Future drug-screening efforts will focus on identifying small molecules, natural products, chemically modified derivatives, peptides, RNA interference molecules, small molecule agonists/inhibitors, and other candidate drugs for mechanism studies, structural optimization, in vivo validation, and drug development assessments. Finally, our findings support the clinical feasibility of targeting CIRBP expression or activity to mitigate chemotherapy-associated cardiotoxicity.

## 5. Conclusions

This study identifies CIRBP as a novel post-transcriptional regulator of ferroptosis resistance in DIC. We demonstrate that CIRBP protects cardiomyocytes by directly binding the 3′ untranslated region of *Slc7a11* mRNA, stabilizing its expression and supporting the SLC7A11/GPX4 antioxidant axis. Loss of CIRBP impairs cystine uptake and glutathione synthesis, leading to ROS accumulation, mitochondrial damage, and ferroptotic cell death. Both in vivo and in vitro experiments confirm that CIRBP overexpression mitigates, while CIRBP knockdown aggravates, cardiac injury under DOX stress.

Clinically, CIRBP may serve as a biomarker or therapeutic target in preventing chemotherapy-induced cardiac damage. Pharmacological strategies to preserve CIRBP function or enhance SLC7A11 translation hold potential for protecting cardiac tissue without impairing anticancer efficacy. Future studies should explore the broader relevance of the CIRBP–SLC7A11–GPX4 axis in other ferroptosis-driven diseases, identify upstream regulators of CIRBP, and investigate CIRBP—targeting small molecules. Overall, our findings offer theoretical innovation and practical avenues for addressing the growing burden of DIC.

## Data Availability

Supplementary data may be found in Supplementary Materials available with the online version of this article. For the original data, please contact yanxiwenny@foxmail.com.

## Supplementary Materials

The following supporting information can be downloaded at: https://www.mdpi.com/article/10.3390/antiox14080930/s1. Figure S1: Subcellular localization of CIRBP and cTnT in cardiomyocytes after DOX treatment. Figure S2: Dual-normalized Western blots confirm genuine DOX-induced suppression of CIRBP (doublet), SLC7A11, and GPX4. Figure S3: DOX differentially modulates SLC7A11, GPX4 and CIRBP levels in cultured cardiomyocytes. Figure S4: Prognostic analysis of CIRBP expression in a clinical cohort. Figure S5: Gene classification and mutational status of CIRBP, SLC7A11, and GPX4 across different patient samples. Figure S6: Identification of CIRBP-related genes and functional enrichment analysis in heart failure.

## Abbreviations

The following abbreviations are used in this manuscript:

3-MA	3-Methyladenine
ANOVA	Analysis of variance
BNP	B-type natriuretic peptide
cTnT	Cardiac troponin T
CCK-8	Cell Counting Kit-8
CIRBP	Cold-inducible RNA-binding protein
CK	Creatine kinase
DAPI	4′,6-Diamidino-2-phenylindole
DCFH-DA	2′,7′-Dichlorofluorescin diacetate
DEGs	Differentially expressed genes
DIC	Doxorubicin-induced cardiotoxicity
DOX	Doxorubicin
ELISA	Enzyme-linked immunosorbent assay
EF	Ejection fraction
Fer-1	Ferrostatin-1
FS	Fractional shortening
GAPDH	Glyceraldehyde-3-phosphate dehydrogenase
GEO	Gene Expression Omnibus
GSH	Glutathione
GSH-Px	Glutathione peroxidase
GO	Gene Ontology
GPX4	Glutathione peroxidase 4
H&E	Hematoxylin and eosin
H9c2	Rat embryonic cardiomyoblast cell line
HW/BW	Heart-weight-to-body-weight ratio
HW/TL	Heart-weight-to-tibia-length ratio
i.p.	Intraperitoneal
IHC	Immunohistochemistry
IF	Immunofluorescence
JC-1	5,5′,6,6′-Tetrachloro-1,1′,3,3′-tetraethyl-benzimidazolyl-carbocyanine iodide
KEGG	Kyoto Encyclopedia of Genes and Genomes
KO	Knockout
LDH	Lactate dehydrogenase
LV	Left ventricle/left-ventricular
MD	Molecular dynamics
MDA	Malondialdehyde
MMP	Mitochondrial membrane potential
mRNA	Messenger RNA
Ns	Not significant
OE	Over-expression
qPCR	Quantitative polymerase chain reaction
RIP	RNA immuno-precipitation
RMSD	Root-mean-square deviation
RMSF	Root-mean-square fluctuation
ROS	Reactive oxygen species
RT-qPCR	Reverse-transcription quantitative PCR
SLC7A11	Solute carrier family 7 member 11
siRNA	Small interfering RNA
SOD	Superoxide dismutase
TEM	Transmission electron microscopy
TUNEL	Terminal deoxynucleotidyl transferase-mediated dUTP nick-end labeling
WGA	Wheat-germ agglutinin
WT	Wild type
