# Supplementary material for: CIRBP Stabilizes Slc7a11 mRNA to Sustain the SLC7A11/GPX4 Antioxidant Axis and Limit Ferroptosis in Doxorubicin-Induced Cardiotoxicity
Source: Antioxidants (Basel). 2025 Jul 29;14(8):930. doi: 10.3390/antiox14080930 (PMC12383039; doi:10.3390/antiox14080930)
Supplement: Supplementary file 1 [file antioxidants-14-00930-s001.zip › antioxidants-3739450-supplementary.pdf]

Supplementary Materials

CIRBP Stabilises *Slc7a11* mRNA to Sustain the SLC7A11/GPX4 Antioxidant Axis and Limit Ferroptosis in Doxorubicin-Induced Cardiotoxicity

Yixin Xie [1,2,] Yongnan Li [2,3,] Yafei Xie [1,2,] Jianshu Chen [1,2,] Hong Ding [1,2] and Xiaowei Zhang [1,2,*]

[1] Department of Cardiology, Lanzhou University Second Hospital, Lanzhou,730000 China; 220220905070@lzu.edu.cn (Y.X.); xieyf20@lzu.edu.cn (Y.X.); chenjsh19@lzu.edu.cn (J.C.); dingh0110@163.com (H.D.)

[2] The Second Clinical Medical College, Lanzhou University, Lanzhou,730000 China; lyngyq2006@foxmail.com

[3] Department of Cardiac Surgery, Lanzhou University Second Hospital, Lanzhou, 730000, China

[*] Correspondence: xwzhang@lzu.edu.cn; Tel.: +86-188-9310-5566

Materials and Methods

Animals

Male adult Sprague Dawley rats were purchased from Lanzhou Veterinary Research Institute, Chinese Academy of Agricultural Sciences (Lanzhou). All animal experiments were performed in accordance with the Guide for the Use and Care of Laboratory Animals (National Academy Press, USA), and all animal experimental protocols were tested in the Guide for the Care and Use of Laboratory Animals of the Second Hospital of Lanzhou University and formally approved by the Institutional Animal Ethics Committee of LUSH (approval number: D2024-191, March 6, 2024).

Generation of *Cirbp*-KO rats

Cirbp-KO rats were produced by using transcription activator-like effector nuclease (TALEN)-based genome editing techniques. TALEN constructs specific for the rat *Cirbp* gene were designed to target its exon (Repeat variable diresidue: TGCCTCTCTCCTGCAGTGGTggtggtaaaggacagggAGACTCAACGATC CCGA; Rat Genome Database ID: 620756; Chr7: 12571310-12575127 reverse strand). TAL Effector Nucleotide Targeter 2.0 (https://tale-nt.cac.cornell.edu/) did not predict off-target site. TALEN expression vector was constructed by incorporating the TALEN construct into pCDNA3.1-TALEN plasmid. The expression vector was tested for integrity and then linearized and transcribed into mRNA and then microinjected into single-cell rat embryos from SD rats. To collect fertilized eggs, fertile female SD rats (8 to 10 weeks) were induced to super-ovulate by intraperitoneal injection of PMSG (30 IU) and hCG (30 IU). These rats were mated with fertile males and those with detected vaginal plug were selected as egg donors. Microinjected embryos were transferred to the oviduct of pseudo-pregnant rats at the day vaginal plug was detected. Genomic DNA of founder rats was extracted from tail biopsies and screened for TALEN-induced mutations at the target site of the *Cirbp* gene. TALEN target site was amplified using the following primers: forward primer 5′-AGATCTCGGAAGGTGAGGCT-3′ and reverse primer 5′-ATCCTCGGGACCGGTTAT CA-3′. Genotyping was performed by the sequencing of the PCR products from tail DNA. The founder

with 7 bp deletion of the gene was chosen for further breeding. Founder rats were mated with wildtype SD males to produce F1 generation. These rats were backcrossed to SD rats for five generations, which were further intercrossed to produce the homozygous line.

Cell viability assay

Cardiomyocytes viability were assessed using Cell Counting Kit-8 (C0038, Beyotime) according to the manufacturer's instructions. Briefly, approximately 5,000 cells (100 μL/well) in the cell suspension were seeded in a 96-well plate. Then the plate was subjected to cold ischemia. The CCK-8 solution (10 μL) was subsequently added to each well, and the cells were incubated for indicated time. Finally, the absorbance was measured at 450 nm using a microplate reader.

Echocardiography

M-mode echocardiography was performed on each rat after transplantation using a small animal echocardiography analysis system (Vevo770, VisualSonics). In brief, rats were anesthetized with isoflurane. The middle part of abdomen was moistened by the ultrasonic coupling agent as previously described (5). The heart rate (HR), left ventricular ejection fraction (EF), left ventricular end-diastolic diameter (LVEDD) and left ventricular end-systolic diameter (LVESD) were measured using a 30-MHz central frequency scan head after capturing the long axis section of the left ventricle. Percent fractional shortening (FS %) was calculated as (LVEDD-LVESD)/LVEDD.

Histological analysis

The harvested cardiac tissues were fixed overnight in 4% paraformaldehyde buffer. Cardiac tissue was paraffin-embedded and sectioned at 4-5 μm. Then, sections were stained with H&E, PTAH and Prussian blue by using commercially available kits (no. DH0003, Leagene; no. DC0001, Leagene; G1424, Solarbio), according to the manufacturer's instructions.

Immunofluorescence staining in cardiac tissues

Briefly, after dewaxing, hydration and antigen retrieval, paraffin-embedded sections were blocked with 5% serum from goat for 2 hours and incubated with primary antibodies overnight at 4° C. Some sections were incubated with IgG to test the specificity of antibodies at same time. Then, sections were stained with fluorochrome- conjugated secondary antibodies for 60 min. Images were captured by a laser-scanning confocal microscope (SP8, Leica). The fluorescence intensity and area were measured by Image J software (version 1.52a). The MPO-positive number was determined by counting puncta of MPO signals in 3 randomly selected views. The primary antibodies were listed in supplementary Supplemental Table 4.

Apoptosis analysis

Tissue sections were prepared after dewaxing, hydrate by graded ethanol and antigen retrieval using citrate. TUNEL staining was performed using an in-situ cell death detection kit (no. 11684795910, Roche) according to the manufacturer's protocol. The apoptotic index was measured by counting puncta of TUNEL signal in 100 randomly selected actinin-positive cells in multiple fields chosen randomly at 400× magnification. Images were captured by a laser-scanning confocal microscope (SP8, Leica).

Immunofluorescence staining in cardiomyocytes

Cardiomyocytes were fixed in 4% paraformaldehyde. Thereafter, cardiomyocytes were blocked with 5% serum from goat for 2 hours and incubated with primary antibodies overnight at 4° C. Some cardiomyocytes were incubated with IgG to test the specificity of antibodies at same time. Then, cardiomyocytes were stained with fluorochrome-conjugated secondary antibodies for 60 min. Images were captured by a laser-scanning confocal microscope (SP8, Leica). The fluorescence intensity and area were measured by Image J software (version 1.52a). The primary antibodies were listed in Supplemental Table 4.

RT-PCR analysis

Total RNA was extracted using the Trizol reagent (no. 15596018, Thermo Fisher Scientific). Reverse transcription was performed using a Primescript RT reagent kit (no. RR047Q, Takara) and RT-PCR was performed using life Technology ABI 7500 System based on SYBR-Green PCR kit (no. A25742, Thermo Fisher Scientific). The △△Ct method was used for the calculation of relative changes in gene expression. The primer sequences for RT-PCR were listed as below: *Cirbp* forward: 5,- GGTCTCAGCTTCGACACCAA -3,; reverse: 5,- TCCCATCCACAGACTTCCCA -3,. *Slc7a11* forward: 5,- CCGAGGACCAAGCTGTCATT-3, and reverse: 5,-GTAAGGGCGTACATCTCCCG-3,. *Gapdh* forward: 5,- CTCTCTGCTCCTCCCTGTTC -3,; reverse: 5,- GCCAAATCCGTTCACACCG -3.

Cytosolic and nuclear proteins extraction

Cytoplasmic and nuclear proteins were extracted from cardiac samples using commercially available assay kits according to the manufacturer's instructions (Minute^{TM}, no. NT-032, Invent).

Western blot analysis

Western blot analysis was performed following a standard protocol. Briefly, total proteins of tissues and cells were extracted using RIPA buffer with protease inhibitors (Roche). For immunoblot analysis, protein samples were resolved by SDS-PAGE and transferred onto nitrocellulose membranes by an iBlot 2 dry blotting system (Thermo Fisher Scientific). Membranes were blocked in 5% non-fat dry milk in TBST (100 mmol/L Tris, pH 7.5, 0.9% NaCl, 0.1% Tween-20) for 1 hour and incubated with primary antibodies overnight at 4°C. Membranes were washed 3 times with TBST and incubated for 1 hour with horseradish peroxidase-labeled secondary antibody at room temperature. After rinsing, enhanced chemiluminescent substrate (no. WBKLS0500, Millipore) was used to develop the membranes. Amersham Imager 600 (GE) was used to capture and analyze the band intensities. The primary antibodies were listed in Supplemental Table 5.

Transmission electron microscope (TEM)

Samples of myocardium (1 mm×1 mm×2 mm) were quickly removed from the left ventricle and immediately fixed with 2.5% glutaraldehyde overnight at 4°C for 48 hours. The fixed tissues were then dehydrated with ethanol and embedded in ultra-thin sections. After the sections were counterstained with uranyl acetate and lead citrate, they were observed under the transmission electron microscope (HT7500, Hitachi, Japan). Five random fields from each sample were screened, and the abnormal mitochondria with mitochondrial shrinkage, mitochondrial exacerbated crista loss and mitochondrial membrane rupture were preserved.

Molecular Docking and Molecular Dynamics Simulations

To further investigate the molecular mechanism of RNA binding to the CIRBP receptor, molecular dynamics simulations of the selected receptor-ligand complexes were performed using the Amber 2020 software package. The protein was parameterized using the AMBER19SB force field, and RNA was parameterized with the RNA.OL3 force field. The TIP3P explicit water model was selected, and the minimum distance between atoms in the receptor and the water box edge was set to 1.0 nm. Based on docking results, sodium or chloride ions were used to neutralize the system's charge. The molecular dynamics simulation workflow included four steps: energy minimization, heating, equilibration, and production dynamics simulation. First, the heavy atoms of the receptor (and RNA) were constrained, and water molecules underwent 10,000 steps of energy minimization (5,000 steps using the steepest descent method and 5,000 steps using the conjugate gradient method). Then, the constraints were released, and the entire system was minimized for another 10,000 steps (5,000 steps using the steepest descent method and 5,000 steps using the conjugate gradient method). During energy optimization, the system was gradually heated to 300 K over a 50 ps period. After heating, the system was equilibrated for 50 ps under the NPT ensemble. Finally, a 100 ns molecular dynamics simulation was performed under the NPT ensemble. The trajectory data was saved every 10 ps and analyzed using the cpptraj module. The binding free energy of the ligand and receptor was calculated using the MMPBSA method.

RMSD (Root Mean Square Deviation) is used to calculate the deviation of atomic positions at a given time from the initial conformation. Monitoring the RMSD of a protein provides insights into the structural conformation of the complex throughout the simulation, helping assess the system's stability and the flexibility of the molecule. A wider RMSD distribution indicates more significant conformational changes of the molecule. From the RMSD plot, we observe that the average RMSD of the complex remains below 3 nm, and the complex reaches dynamic equilibrium around 80 ns. However, the RMSD fluctuation of CIRBP protein is relatively small, which could be due to the protein being embedded within the RNA cavity, limiting its conformational changes. The RNA exhibits larger conformational changes, likely related to the initial RNA model's defects, as effective methods for predicting RNA structure are still lacking. As seen in Fig. 1, CIRBP continues to form multiple hydrogen bonds with RNA residues at A-108, A-105, C-107, C-24, C-16, C-17, and U-18. Additionally, U-18 of RNA can form an electrostatic interaction with ARG-46, suggesting that after dynamic adjustments, RNA still stably binds to the protein, forming a stable complex.

RMSF (Root Mean Square Fluctuation) is used to characterize the conformational changes of each amino acid in the protein chain during the simulation, with peaks indicating the regions with the largest fluctuations. A larger RMSF value indicates greater conformational fluctuations of the amino acids and greater flexibility. According to the RMSF plot, only a small portion of the amino acids in the protein-RNA complex exhibit significant conformational changes (e.g., in the 150-200 region). This is mainly because the residues in this region are located in the hinge area of the complex, which has relatively high flexibility, allowing these residues to undergo conformational changes during the simulation. However, most of the amino acids show conformational changes within an acceptable range.

To analyze the relative compactness and stability of the complex induced by binding at primary and secondary sites, we measured the radius of gyration (Rg) of the target protein, which calculates the

mass-weighted distance between the receptor atoms and the center of mass. Rg evaluates the compactness of the protein structure: a lower Rg value indicates a more compact protein, while a higher Rg value suggests greater conformational entropy and disorder. From the Rg plot, we can see that the Rg of CIRBP protein slightly increases, becoming slightly higher than that of the initial complex state. This could be due to unreasonable internal contacts in the initial complex model. After molecular dynamics adjustments, new internal interactions form, reaching a new equilibrium.

For a more intuitive understanding of the protein-RNA binding, we tracked the number of hydrogen bonds between CIRBP protein and RNA throughout the simulation. From the hydrogen bond network diagram, it is clear that RNA consistently forms over twenty hydrogen bonds with the amino acids in the protein's pocket. These hydrogen bonds play a critical role in stabilizing the RNA-protein interaction. Moreover, according to Table 1, the hydrogen bonds formed between GLN-25 and A-105, ARG-46 and U-18, GLN-32 and C-107, ARG-46 and C-17, GLN-45 and C-16, and GLN-32 and A-108 account for 70% of the total hydrogen bonds during the simulation, highlighting their significant role in stabilizing the protein-RNA complex.

The binding free energy is a fundamental tool for analyzing changes in the ligand binding mode by measuring the thermodynamic properties of the ligand. Negative binding free energy values (ΔGbinding energy) indicate system stability, while positive values suggest instability. Van der Waals interactions play a major role in stabilizing RNA, followed by electrostatic interactions. The binding free energy of RNA and CIRBP protein is -244.957±9.241 kcal/mol, with electrostatic interactions dominating (-3404.975±153.939 kcal/mol). This suggests that the protein remains stably bound in the RNA cavity, with strong electrostatic interactions with surrounding residues. Additionally, the protein's ability to stay within the RNA cavity contributes significantly to the van der Waals interactions, which also play an important role in stabilizing the protein (-134.437±8.395 kcal/mol).

Supplemental Figures

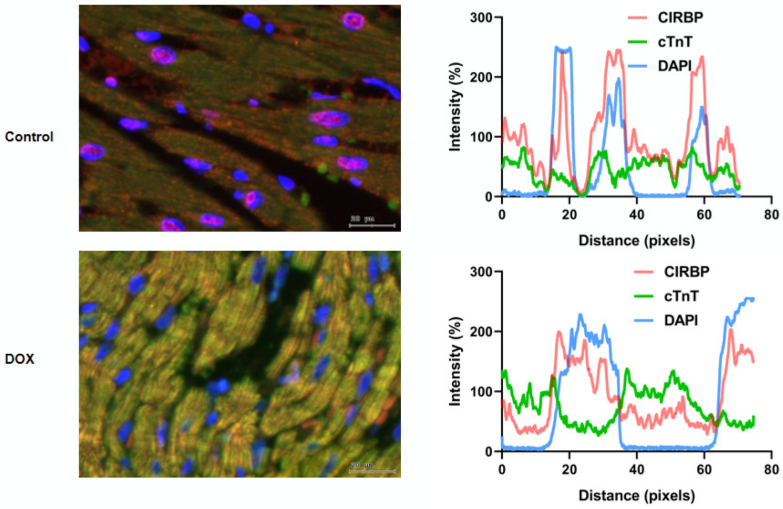

Supplemental Figure 1. Subcellular localization of CIRBP and cTnT in cardiomyocytes after DOX treatment. Representative confocal images of cardiomyocytes stained for CIRBP (red), cTnT (green), and nuclei (DAPI, blue) in the control (top) and DOX-treated (bottom) groups. Scale bar = 20 µm. The intensity profiles (right) show the distribution of CIRBP, cTnT, and DAPI along a selected line in the images. The intensity data are expressed as a percentage of the maximum intensity across the images for each marker, with CIRBP and cTnT showing different patterns of localization before and after DOX treatment.

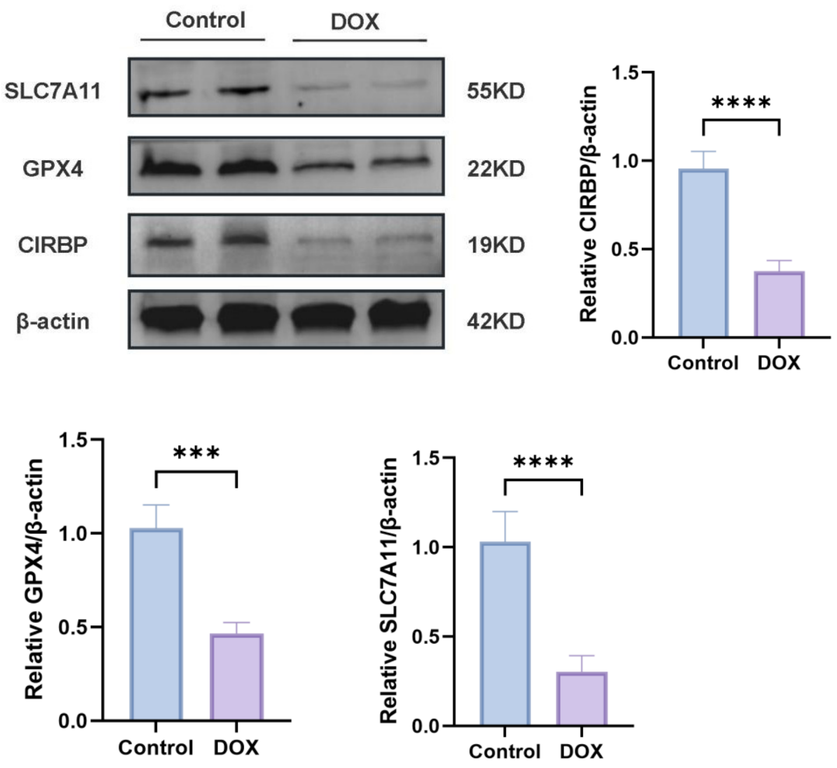

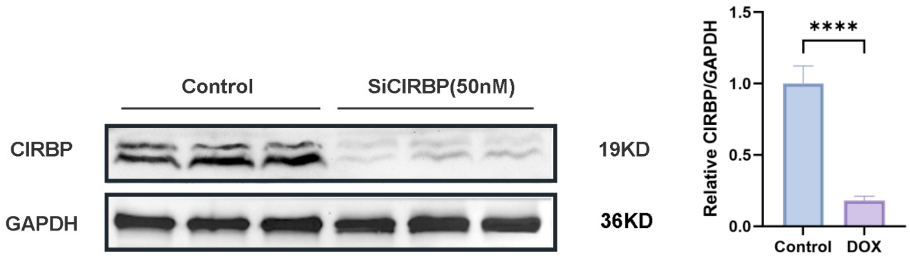

Supplemental Figure 2. Dual-normalised Western blots confirm genuine DOX-induced suppression of CIRBP (doublet), SLC7A11, and GPX4. The consistent down-regulation after dual normalisation demonstrates that the observed suppression is a genuine biologic al effect rather than a loading artefact. Knock-down of CIRBP caused a parallel reduction in both upper and lower CIRBP bands, indicating that the doublet originates from CIRBP-derived species rather than non-specific signals. Representative Western blots showing SLC7A11 (55 kDa), GPX4 (22 kDa), CIRBP (19 kDa), and β-actin (42 kDa) in lysates from control cells and cells treated with DOX (1 µM, 24 h). To avoid DOX-induced fluctuations in glycolytic housekeeping proteins such as GAPDH, β-actin was used as an additional internal control under the same protocol.Bar graphs depict the relative expression of CIRBP, GPX4, and SLC7A11 after dual normalisation to GAPDH and β-actin; each dataset confirms a robust reduction of the three targets following DOX exposure. Representative Western blot illustrating efficient CIRBP knock-down in H9C2 cells. Cells were transfected with either control siRNA or siCIRBP (50 nM) for 48 h, and lysates were probed for CIRBP (19 kDa); GAPDH (36 kDa) served as the loading control. Data are presented as mean ± SD (n = 3 independent experiments). Statistical significance was assessed by unpaired two-tailed Student's t-test (*** $p < 0.001$; **** $p < 0.0001$).

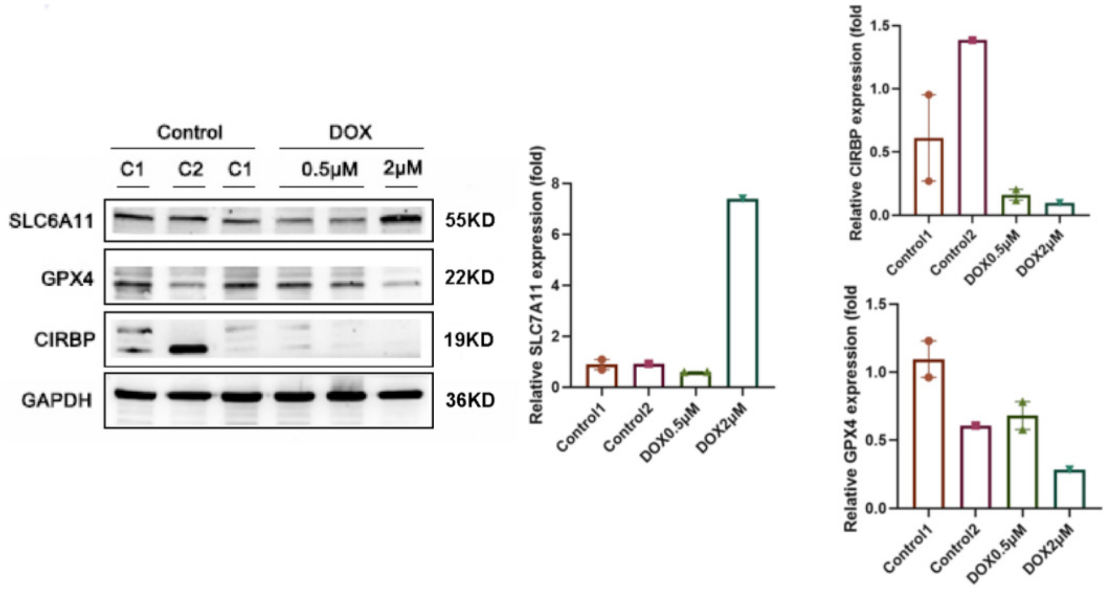

Supplemental Figure 3. DOX differentially modulates SLC7A11, GPX4 and CIRBP levels in cultured cardiomyocytes. Representative Western blot images show the expression of SLC7A11 (55 kDa), GPX4 (22kDa), and CIRBP (19 kDa) in cardiomyocyte lysates from two independent controls (C1, C2) and after 24 h exposure to DOX (0.5 µM or 2 µM). GAPDH (36 kDa) served as the loading control. Bar graphs depict densitometric quantification of each protein,

normalized to GAPDH and expressed relative to the mean of the control group (mean ± SD, n = 3). SLC7A11 exhibited a marked transient up-regulation at 2 µM DOX—consistent with an acute stress response—whereas CIRBP and GPX4 were progressively down-regulated with increasing DOX concentration. Prolonged DOX exposure (not shown) eventually led to a decline in SLC7A11 expression, indicating that the initial induction is not sustained.

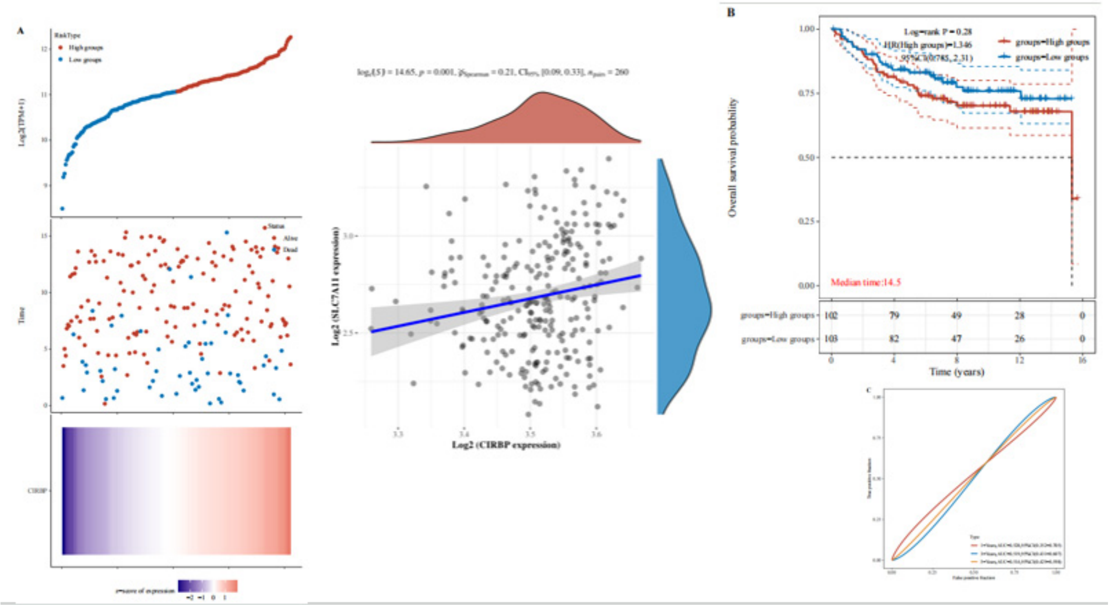

Supplemental Figure 4. Prognostic analysis of CIRBP expression in a clinical cohort. (A) The distribution of CIRBP expression across different patient groups. The top plot shows the cumulative distribution of CIRBP expression (log2 scale) for high (red) and low (blue) CIRBP expression groups. The middle plot displays the time-to-event data for each patient, with high and low expression groups marked in red and blue, respectively. The bottom heatmap visualizes the expression pattern of CIRBP across the cohort, with color intensity reflecting expression levels. The statistical significance of the association between CIRBP expression and patient outcomes was assessed using the log-rank test (p = 0.001), and a positive correlation was found between CIRBP expression and survival time (log2 scale) with a regression coefficient of 0.21. (B) Kaplan-Meier survival curves comparing overall survival between the high and low CIRBP expression groups. The high CIRBP expression group (red) showed a non-significant trend toward better survival (log-rank p = 0.28). The median survival for the high CIRBP group was 14.5 years, while the low CIRBP group had a median survival of 10.5 years. (C) The concordance index (C-index) was used to assess the discriminatory ability of CIRBP expression as a prognostic marker. A C-index value of 0.62 indicates moderate prognostic potential for CIRBP expression in predicting patient outcomes.

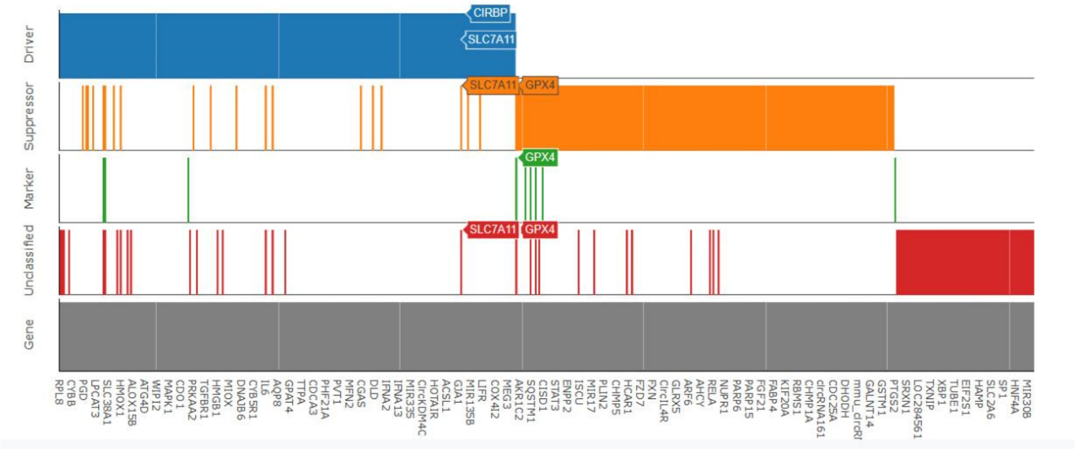

Supplemental Figure 5. Gene classification and mutational status of CIRBP, SLC7A11, and GPX4 across different patient samples. The top panel categorizes genes as Driver (blue), Suppressor (orange), Marker (green), and Unclassified (red). The specific genes of interest, CIRBP, SLC7A11, and GPX4, are highlighted in the top rows within the appropriate categories. The horizontal bars represent the presence of mutations in these genes across different patient samples (listed in the bottom row). Genes classified as "Driver" are associated with tumorigenesis, while "Suppressor" genes are linked to tumor suppression. "Marker" genes are indicative of disease progression or therapeutic response, while "Unclassified" genes require further investigation. The colors within the rows represent the mutation type or status for each gene in individual patient samples.

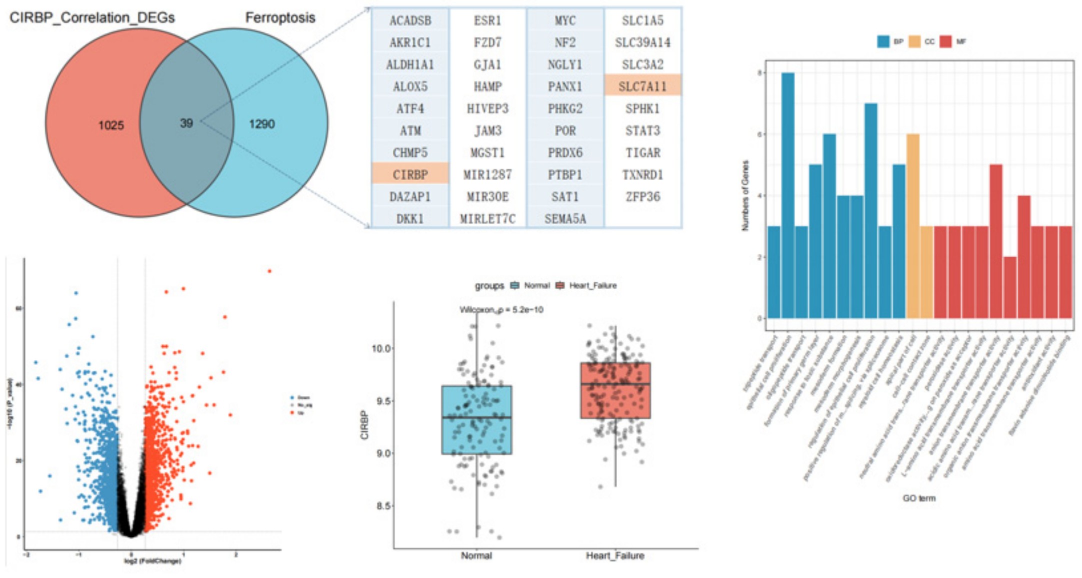

Supplemental Figure 6. Identification of CIRBP-related genes and functional enrichment analysis in heart failure. (A) Venn diagram showing the overlap between CIRBP-correlated differentially expressed genes (DEGs) and ferroptosis-related genes. The table lists genes that are common to both categories, with CIRBP, SLC7A11, and SLC3A2 highlighted in orange.(B) Volcano plot depicting the differential expression of genes between normal and heart failure samples. Upregulated genes are shown in red, and downregulated genes are shown in blue, with

non-significant genes in black.(C) Boxplot illustrating the expression levels of CIRBP in normal and heart failure groups, with a significant difference determined by the Wilcoxon test (p = 5.2e-10).(D) Gene Ontology (GO) analysis showing the functional classification of differentially expressed genes in heart failure. The bar plot represents the number of genes associated with biological processes (BP), cellular components (CC), and molecular functions (MF).
